# Reconstructing developmental lineages: a retrospective approach using somatic mutations and variant allele frequency

**DOI:** 10.3389/fgene.2025.1761810

**Published:** 2026-01-09

**Authors:** Mahnoor Sajjad, Seong Gyu Kwon

**Affiliations:** Department of Anatomy and Convergence Medical Science, College of Medicine, Institute of Medical Science, Gyeongsang National University, Jinju, Republic of Korea

**Keywords:** embryogenesis, lineage tracing, lineage tree, somatic mutation, variant allele frequency (VAF), whole-genome sequencing (WGS)

## Abstract

Somatic mutations accumulate during the first zygotic division and continue throughout an organism’s lifespan. The characteristics and frequency of these mutations are contingent on developmental timing and tissue type, giving rise to somatic mosaicism, defined as the presence of unique genomic alterations across different cells. They serve as endogenous cellular barcodes, enabling detailed reconstruction of cell lineages and clonal dynamics. Although lineage tracing techniques have advanced from early microscopic observation and dye staining to the introduction of artificial barcodes via gene editing, owing to ethical considerations, such genetic manipulations in human developmental research are unavailable. Therefore, spontaneously arising somatic mutations are the most suitable strategy for tracing human lineages. Current approaches can be broadly categorized into two strategies: (i) high-resolution methods, including single-cell clonal expansion or laser-capture microdissection, which construct precise phylogenetic trees based on shared mutation profiles; and (ii) bulk sequencing methods, which infer lineage proximity by comparing variant allele frequencies across samples. As more lineage-tracing studies are being conducted focusing on a wider variety of organs, the integration of such data will make it possible to discover the general principles governing human development. This review highlights how the concept of somatic mutations has been applied across diverse biological contexts and discusses the insights and common principles that can be drawn from these findings.

## 1 Introduction

During somatic cell division, errors that occur during DNA replication can result in permanent changes in the DNA sequence, known as somatic mutations (Lodato and Vijg, 2022). These somatic mutations can be induced by both internal and external factors, including replication errors and environmental effects. These mutations are permanently “scarred” in the DNA of the daughter cells if not corrected (Loeb and Cheng, 1990).

These somatic mutations accumulate continuously from the first division of the zygote throughout the growth of the organism (Mohiuddin et al., 2022), and their characteristics and frequency vary depending on developmental timing and tissue type in terms of internal factors (Kim et al., 2022).

Consequently, individual cells in the same organism have distinct somatic mutations, making cell populations genetically heterogeneous. This phenomenon is referred to as somatic mosaicism, which describes the presence of unique genomic alterations in different tissues or cells depending on the timing and location of somatic mutations that occur after zygote formation (Freed et al., 2014).

Somatic mosaicism indicates that mutations at the single-cell level can be distributed across multiple cells and tissues within an organism. Recent studies have shown that somatic mutations can occur in different ways and in different tissues at various stages of development (De, 2011).

Somatic mutations include various genetic alterations that occur in cells after fertilization. The types of somatic mutations include single-nucleotide variants (SNVs), small insertions and deletions (indels), and large structural variations, such as deletions, duplications, and translocations, as well as copy number variations (Pleasance et al., 2010).

In clinical research, particularly in cancer genetics, somatic mutations are often classified based on their functional roles. For example, somatic variants can be categorized as oncogenic, likely oncogenic, variants of uncertain significance, likely benign, or benign, depending on their potential to drive disease processes, such as tumorigenesis (Horak et al., 2022).

Somatic mutations are the key drivers of the initiation and progression of many cancers. Somatic mutations in EGFR and KRAS are key drivers of lung cancer. EGFR mutations are associated with a favorable response to EGFR tyrosine kinase inhibitors (TKIs) and improve overall survival. In contrast, KRAS mutations are associated with reduced responsiveness to EGFR-TKIs and predict shorter survival in patients with advanced lung adenocarcinoma (Johnson et al., 2013; Wang et al., 2024). In breast cancer, driver mutations frequently occur in genes such as PIK3CA and TP53. PIK3CA mutations are most common in estrogen receptor (ER)-positive breast cancers, whereas TP53 mutations are predominant in ER-negative cases. TP53 mutations are associated with poorer prognosis in ER-positive metastatic breast cancer, whereas in ER-negative cases, TP53 mutations may have a protective effect (Kim et al., 2017; Rajendran and Deng, 2017). For colorectal cancer, the most recurrent driver mutations have been found in APC, TP53, and KRAS. These mutations contribute to clonal expansion and chemoresistance of cancer cells. Concurrent KRAS and TP53 mutations are strongly associated with poor response to standard chemotherapy, increased risk of recurrence and metastasis, and worse overall prognosis (Matas et al., 2022; Tang and Fan, 2024). Mutations in BRCA1/2 and TP53 are important for ovarian cancer. BRCA1/2 mutations are associated with increased sensitivity to platinum-based chemotherapy and PARP inhibitors, resulting in improved survival. However, TP53 accumulation can modify the prognostic impact of BRCA1 mutations, particularly in high-grade serous ovarian carcinomas (Pennington et al., 2014; Rzepecka et al., 2017).

Recent studies revealed that somatic mutations are also present in normal tissues, with their accumulation beginning from the first cell division after conception (Rahal et al., 2024). The number of accumulated somatic mutations varies significantly among different tissue types and species (Cagan et al., 2022). For example, mutation rates are higher in tissues such as the colon and skin than in germline cells or the brain (Werner and Sottoriva, 2018).

The accumulation of somatic mutations is influenced by various factors, including environmental exposure (such as tobacco smoke or UV light), cell division rates, and DNA repair efficiency, leading to individual- and tissue-specific differences in mutation burden (Ren et al., 2022). The pattern of somatic mutation accumulation is known as a mutational signature. A comprehensive analysis of mutational signatures in normal tissues has shown that mutational processes similar to those found in cancer cells are also found in normal cells (Yaacov et al., 2023; Boysen et al., 2025).

In addition to analyzing somatic mutations in diseases or cancers, somatic mutations can also be used as natural barcodes for retrospective cellular lineage tracing (Behjati et al., 2014). Since somatic mutations occur at random sites across the genome, they act as distinct cellular identifiers, enabling the detailed tracing of cell lineages and clonal dynamics (Park et al., 2021). Multiple studies have applied lineage tracing based on somatic mutations in diverse tissues to discover lineage relationships between cells and tissues from various organs (Bae et al., 2018; Coorens et al., 2021a; Park et al., 2021; Spencer Chapman et al., 2021).

Retrospective lineage tracing using somatic mutations fundamentally relies on the fact that starting from the zygote, mutations accumulate uniquely as cells divide. As cells continuously divide throughout their lifetime, they accumulate genetic mutations, enabling the inference of their division history. In other words, the presence or absence of shared somatic mutations among cells allows for the complete reconstruction of their developmental trajectories (Choi et al., 2023).

As research on lineage tracing using somatic mutations has increased, there is a growing need for techniques to construct phylogenetic trees based on somatic mutations identified in various tissues from a single organism. However, differences in variant filtering criteria and tree construction algorithms among the studies have been identified as limitations. A recent study presented detailed guidelines for building phylogenetic trees using somatic mutations, suggesting that these could serve as standards for lineage-tracing research (Coorens et al., 2024).

The variant allele frequency (VAF) concept has been applied to somatic mutations in lineage-tracing studies. The VAF derived from bulk sequencing data, where cells of multiple origins are mixed, reflects the relative prevalence of a given somatic mutation within the sample (Dou et al., 2018). In other words, VAF serves as an indicator of the contribution of cells with a particular somatic mutation to the cell population in the bulk tissue.

In this review, we analyze studies that utilized somatic mutations for developmental lineage tracing. We highlight how this concept has been applied across diverse biological contexts and discuss the insights and common principles that can be drawn from these findings.

## 2 Methods for developmental lineage tracing

### 2.1 Early studies

One of the earliest documented studies on developmental lineage tracing was conducted by Charles Otis Whitman in the late 19th century. Whitman observed and mapped the fate of each cell during leech embryonic development (Whitman, 1878). His work suggests that the fate of cells in the developmental stage is determined in the early cleavage stage and is not a stochastic process as previously thought.

Sulston and his colleagues traced the complete lineage of every cell in *Caenorhabditis elegans* from fertilization to adulthood (Sulston et al., 1983). This was a milestone study in developmental biology, and the authors reported when and how the fate of cells during early development was determined. Additionally, they found that the cells disappeared naturally, proposing the idea of programmed cell death.

Given the limitations of microscopic observation of cells, studies have been conducted to analyze cell fate using vital dyes. The dye must effectively stain the target cells without being harmful to the cells. This method has been used in studies that traced the development of *Xenopus* up to the 32-cell stage (Vogt, 1929) and in a study that determined the fate map of the zebrafish neural plate (Woo and Fraser, 1995). However, the use of dyes has disadvantages: they can leak into adjacent cells, and their concentration is diluted with each cell division, leading to a decrease in accuracy.

### 2.2 Implications of genetic tools

The rapid advancement in genetic tools since the 1990s has improved approaches to lineage tracing, enabling more precise tracking of developmental processes than in previous studies. The Cre-Lox-based technique is the most widely used for modern genetic lineage tracing. The Cre-loxP system regulates the tissue-specific activation or inhibition of gene function, enabling progenitor cells to produce descendant cells marked by reporter genes that serve as permanent and inheritable genetic barcodes (Wang et al., 2023). Several studies have been conducted on lineage tracing across different tissues using this method. In the lung, it has been revealed how club cells regenerate ciliated cells following airway injury (Rawlins et al., 2009). Additionally, this approach has revealed how epicardial progenitors contribute to cardiomyocyte formation during heart development (Zhou et al., 2008). Similar methodologies have been applied to investigate the origin of neural cells (Adameyko and Lallemend, 2010). Taken together, Cre-lox-based lineage tracing is a powerful tool capable of tracking cell fate across a broad timeline ranging from early developmental stages to specific cell populations at later developmental time points.

Similar to the Cre-loxP system, CRISPR-Cas9-based lineage tracing introduces mutations that act as barcodes for the reconstruction of cellular phylogenies. This approach has been applied to track developmental processes in animals. For instance, in zebrafish, CRISPR-Cas9 has been used to create mutations in early embryos, allowing researchers to build comprehensive lineage trees that reveal the relationships between different cell types as an organism develops. Furthermore, in a study on pancreatic cancer metastasis, researchers used the CRISPR-Cas9 tool macsGESTALT to create unique genetic markers for individual cancer cells in a mouse model. This enabled them to track how thousands of these single cells spread throughout the body to form new tumors in different locations (Simeonov et al., 2021).

Taken together, genetic tools have significant advantages for lineage tracing by providing permanent and heritable markers, unlike the traditional methods of observing embryos under a microscope or using dyes.

### 2.3 Retrospective lineage tracing using somatic mutations

Despite the advantages of genetic tools, their application for prospective lineage tracing in humans is not feasible. There are ethical concerns about intentionally introducing permanent genetic variations into human participants, even for research purposes (Almeida and Ranisch, 2022). Therefore, alternative methods that do not raise ethical concerns are required for studying lineage tracing in humans.

**FIGURE 1:**
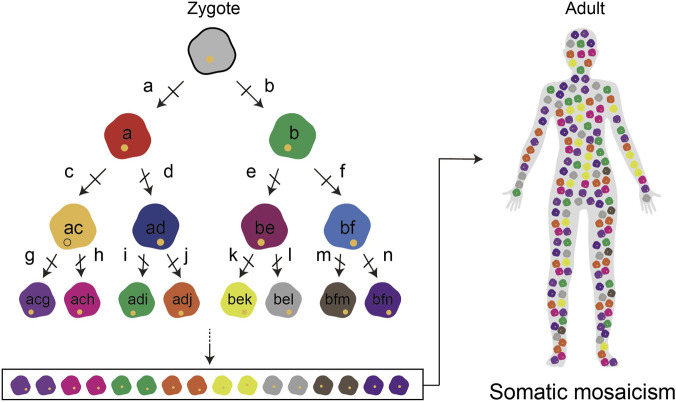
Somatic mutations result in somatic mosaicism in adults Somatic mutations, occurring at each cell division, are represented as ‘a’ through ‘n.’ The final combination of mutations provides each cell with a unique set of characteristics. Consequently, the distinct properties of adult tissues show a mix of different cells, referred to as somatic mosaicism.

As described above in the Introduction, somatic mutations occur naturally during cell division, and each mutation acts as a specific DNA barcode that accumulates in different patterns in each cell. The core principle is that as an embryo develops, distinct barcode patterns are introduced into the genome of each daughter cell in the form of mutations. These mutations accumulate with each subsequent cell division. Consequently, by the time development is complete, the DNA of cells in the adult organism will be ‘scarred’ with a unique and diverse pattern of these mutational barcodes, reflecting their lineage history (Figure 1).

Since somatic mutations are present in every cell in a unique pattern, single-cell analysis enables the most accurate reconstruction of cell lineage tracings. Genomic DNA in single cells can be amplified for whole-genome sequencing (WGS) using whole-genome amplification (WGA). Although WGA is a powerful tool for discovering somatic mutations in a single cell, it creates a large number of errors, including false mutations (Dou et al., 2018) and biased amplification of two alleles (Huang et al., 2015).

Cell proliferation through cell division is a form of DNA amplification. The key difference between WGA techniques is that one occurs within the cell, whereas the other is an artificial process outside the cell. The accuracy of intracellular DNA replication with repair mechanisms is considerably higher than that of WGA (more than 1,000 times) (Youk et al., 2021). Therefore, to accurately identify somatic mutations in a specific cell, clonal expansion of single cells can be used to obtain sufficient DNA for analysis. However, not all cells, especially differentiated cells, are difficult to culture *in vitro* to acquire sufficient amounts of DNA (Bukowy-Bieryłło, 2021). Therefore, the cell types suitable for lineage tracing using this method are limited. Various alternative methods have been developed to extend the application of somatic mutation-based lineage tracing to a broad range of tissues.

Certain stem cells grow clonally in specific compartments (niches) *in vivo* (Simons and Clevers, 2011; Blanpain and Simons, 2013). This process is usually observed in the intestinal crypts (Snippert et al., 2010). A recent study quantitatively confirmed the occurrence of clonal proliferation. The clonal expansion of cells has been observed primarily in the glands within the digestive tract (Moore et al., 2021). Cell populations formed by the clonal growth of a specific stem cell can theoretically be considered analogous to populations generated through single-cell clonal expansion, as described earlier. Once clonal populations are identified under a microscope, a specialized technique called laser-capture microdissection (LCM) can be used to physically isolate the niche region for WGS analysis (Ellis et al., 2021). Using this method, the quantity and quality of the DNA extracted for WGS are poorer than those with single-cell clonal expansion but still demonstrate higher levels of quantity and quality than in WGA (Youk et al., 2021).

To overcome the limitations of WGA, a recent method called primary template-directed amplification (PTA) enables the uniform amplification of DNA from single cells or small numbers of cells, allowing for the accurate detection of somatic mutations (Gonzalez-Pena et al., 2021). Therefore, PTA can be used to detect somatic mutations in tissues where single-cell clonal expansion is impossible and the *in vivo* niche is difficult to identify, such as the brain (Kalef-Ezra et al., 2024).

Ultimately, various approaches for inferring developmental processes in normal cells share the common goal of accurately detecting somatic mutations embedded in the DNA for retrospective lineage tracing.

## 3 Lineage tracing studies in humans

### 3.1 Building direct lineages with clonally expanded cell populations

Most lineage-tracing studies have been conducted by constructing phylogenetic trees that focus on specific organs. A representative example is the collection of skin fibroblasts from donated cadavers, followed by single-cell clonal expansion and phylogenetic reconstruction (Park et al., 2021). In terms of scale, the cited study by Park et al. surpasses previous studies by the number of people included (five cadavers) and by collecting more samples (more than 300 samples). In their study, samples were collected from multiple anatomical sites on the body. Based on the somatic mutation patterns identified in each sample, phylogenetic trees were reconstructed to relate the anatomical location to lineage relationships. Additionally, they used simulations to determine the cell division stage at which the epiblast and trophoblast lineages were segregated from the zygote. The most significant finding of this study is that identifying such a cell division stage demonstrates the possible reason why the two daughter cells arising from the first division contribute to the whole body in different proportions, which is called “asymmetric distribution.” Subsequently, this hypothesis was experimentally verified by detailed observations of the actual division of embryos (Junyent et al., 2024).

The greater the number and diversity of samples analyzed, the more precisely the later cell division stages can be inferred. Therefore, this study provides an important benchmark for future lineage-tracing analyses.

Concurrently, phylogenetic trees were reconstructed from various tissues in other studies. Using LCM, this research enabled lineage tracing through somatic mutation analysis in a diverse range of cells, overcoming the prior limitation of single-cell clonal expansion techniques, which were restricted to specific cell types (Coorens et al., 2021a). The study also confirmed the phenomenon of asymmetric distribution in cell lineages and observed when the fates of the trophectoderm and inner cell mass were determined. These findings provide crucial clues for tracking embryonic development in humans.

Additionally, some studies have analyzed the lineage of specific organs or tissues. Lodato et al. examined somatic mutations using single-cell sequencing of neurons from three normal brains (Lodato et al., 2015). They constructed a phylogenetic tree and identified lineage distances between different brain regions. Bae et al. analyzed somatic mutations in single-cell clonal expansion samples from the forebrains of three fetuses (Bae et al., 2018). They found 200−400 SNVs in each cell and constructed small lineage trees based on the sharedness of the mutations. It is noteworthy that single-cell clonal expansion technology has enabled the discovery of more accurate mutations than single-cell sequencing used in previous brain studies.

Lineage tracing techniques have also been applied to the study of hematopoiesis. Hematopoietic stem cells (HSCs) and progenitor cells (HPCs) were isolated from the bone marrow. Following single-cell clonal expansion, somatic mutations were analyzed to construct a phylogenetic tree. This tree was subsequently used to define the lineage relationship between the 2 cell types and estimate the effective population size contributing to blood cell production (Lee-Six et al., 2018). Similarly, other studies constructed lineage trees from HSCs and HPCs and analyzed the signatures of accumulated somatic mutations to investigate their association with leukemia (Osorio et al., 2018). The group led by Peter J. Campbell has conducted extensive research using lineage tracing in hematopoietic cells to investigate a wide range of associated biological phenomena. They collected a substantially larger number of cells from fetal hematopoietic organs than previous studies. A phylogenetic tree constructed from these cells was used to estimate the divergence time between the embryonic and extraembryonic lineages (Spencer Chapman et al., 2021). This finding is consistent with results previously observed in fibroblasts (Park et al., 2021). The similarity in results, even when constructing independent lineage trees from different tissues, indicates that most cells share a common developmental fate during early cell division. Further investigations involved the collection of hematopoietic cells from donors of various ages to construct lineage trees (Mitchell et al., 2022). These trees were used to evaluate changes in clonal structure and emergence timing of driver mutations. A key finding was that the hematopoietic clonal diversity diminished with age. Beyond lineage tracing, the same research group examined how clonal diversity is altered in patients after hematopoietic cell transplantation (Spencer Chapman et al., 2024). Finally, the study was extended to mice by performing lineage tracing using a similar method and cell type (Kapadia et al., 2025). This comparative analysis with human data demonstrates the potential for future applications in other species.

Studies of lineage tracing in other organs are limited. For example, Tim et al. used LCM to sample tissues from the placenta (Coorens et al., 2021b) and gastric epithelium (Coorens et al., 2025) and performed lineage tracing on a small scale. Technological advancements in single-cell manipulation are expected to enable the application of lineage tracing to a wide variety of organs.

### 3.2 Inferring lineage relationships from bulk sequencing samples

**FIGURE 2:**
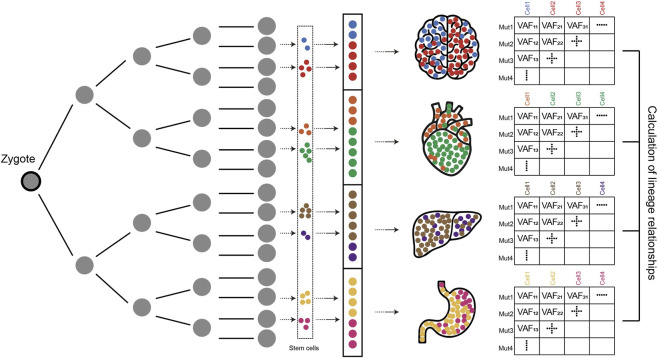
Calculation of lineage relationships with VAFs Cells carrying different somatic mutations eventually form specific organs at various developmental stages. Bulk tissues are composed of stem cells of multiple origins. By analyzing the relationships between mutations and their respective VAFs, the lineage relationships between different organs and tissues can be estimated.

Using single-cell clonal expansion and LCM technology, we can estimate lineage relationships at the single-cell level or at a similar resolution. However, when single-cell culture is impossible or tissue characteristics prevent LCM from isolating clonal populations, indirect lineage tracing is possible by analyzing the bulk tissue. Unlike single-cell clonal expansion samples, in bulk samples, somatic mutations exist not in a presence or absence state but as values of varying degrees, and the metric representing these values is the VAF (Moeller et al., 2023). Bulk samples are collections of single cells of multiple origins containing diverse combinations of somatic mutations, and at some point during development, stem cells expand to predominantly form specific tissues or organs. Therefore, the lineage relationship between the bulk samples was estimated by comparing the similarity of VAFs of the somatic mutations (Figure 2).

One study analyzed somatic mutations in blood using bulk WGS, estimating the developmental stage at which each mutation occurred based on its VAF. This study revealed the unequal contribution of early embryonic cells to adult somatic tissues (Ju et al., 2017). Notably, this finding supports a conclusion similar to that reported by Park et al. (2021) using a single-cell clonal expansion analysis.

In 2021, two similar studies identified somatic mutations in multiple organ tissues through bulk WGS and target sequencing, and estimated developmental lineage tracing (Bizzotto et al., 2021; Fasching et al., 2021). A consistent finding from both studies was the asymmetric contribution of early progenitors to extraembryonic tissues, three germ layers, and organs.

Unlike the studies described above, a recent study provided detailed analyses of the developmental processes of a specific organ. Bulk WGS was conducted on tissue samples from multiple regions of the left and right brain to identify somatic mutations (Breuss et al., 2022). Pearson’s correlation coefficients were calculated for these mutations using their VAFs across the collected samples, followed by clustering analysis. The results demonstrated location-specific grouping, where mutations from distinct brain regions were clustered together. An important finding of this study is that the migration of brain precursor cells across the anterior-posterior and ventral-dorsal axes is constrained after a specific time during development, which was inferred from the distinct spatial distribution of somatic mutations. Furthermore, we identified somatic mutations in single nuclei via amplicon sequencing to confirm the lineage relationships. However, it still has limitations in that it does not demonstrate the same level of accuracy as techniques such as clonal expansion or LCM.

Bulk WGS enables the identification of somatic mutations and the inference of lineage relationships regardless of the tissue type. However, this approach has a limitation in that mutations acquired later in development often exhibit low VAFs, making their detection challenging (Menon and Brash, 2023). Low-VAF mutations are supported by few reads that contain mutations, and it is difficult to distinguish true variants from technical artifacts without harsh filtering methods (Huang and Lee, 2021). To overcome these limitations, technologies for detecting low-VAF variants, such as duplex sequencing (Kennedy et al., 2014) and NanoSeq (Abascal et al., 2021), are being developed. Duplex sequencing tags both DNA strands to generate a consensus. By validating mutations on both strands, it eliminates artifacts and enables the detection of ultra-low-VAF variants. NanoSeq improves duplex consensus by minimizing library preparation artifacts. With an ultra-low error rate (<5 per billion), it detects even single-molecule mutations with high specificity.

## 4 Lineage tracing studies in other species

Somatic mutation-based lineage tracing is mostly used in human studies as it circumvents ethical concerns. Concurrently, this methodology can also be applied to animal models to provide a comparative framework for understanding developmental processes in humans.

The first organismal lineage-tracing study at the single-cell level was conducted using mouse-derived organoids (Behjati et al., 2014). Although the scale of this study was small, it revealed that the two daughter cells from the initial zygotic division were unequally distributed in the adult tissue, a finding consistent with subsequent studies.

The application of single-cell clonal expansion to lineage tracing has been extended to pigs (Kwon et al., 2024). Parallel to the findings of human studies, this study demonstrated an asymmetric contribution from early developmental cells in pigs.

In another study, bulk WGS was applied across multiple mouse organs to investigate lineage relationships by analyzing somatic mutations and their VAFs (Uchimura et al., 2022). Their study demonstrated that for VAF-based lineage tracing, it is crucial to obtain accurate VAFs through high-coverage WGS, and VAF values must be acquired from multiple tissue sources.

Lineage tracing in non-human animals can facilitate comparative analysis of mammalian development and validate the utility of experimental disease models for human pathologies.

## 5 Discussion

Somatic mutations occur during development and leave permanent genomic barcodes across descendant cells. This gives rise to somatic mosaicism and intra-individual genetic heterogeneity in normal tissues. Therefore, mutational fingerprints act as permanent records of an organism’s developmental history.

Lineage tracing methods using somatic mutations are employed to estimate human development when invasive techniques such as gene editing cannot be used. The application of WGS to single-cell clonal expansion samples has enabled the reconstruction of lineage trees at single-cell resolution, offering detailed insights into the developmental history of organisms and specific tissues. A prominent finding from these analyses is the unequal contribution of cells from the early developmental stages to adult tissue composition.

The reconstruction of accurate lineage trees not only explains the developmental processes of normal tissues but also provides crucial insights into pathogenesis, enabling the inference of when disease-initiating mutations occur. This methodology has been extensively applied to hematopoietic cells to determine the developmental timing of various mutations associated with hematological malignancies. This approach has significant potential for identifying the developmental origins of cancers and genetic disorders across diverse tissue types.

Bulk WGS is not suitable for reconstructing formal lineage trees but has broad applicability, enabling the estimation of lineage relationships from nearly all tissue types.

This review surveyed major studies on lineage tracing in normal tissues using somatic mutations, and we observed that research focused on specific organs is not yet widely diversified. As more lineage-tracing studies are being conducted focusing on various organs, the integration of such data will make it possible to discover the general principles of human development.

In conclusion, analysis of lineage relationships at the cellular and tissue levels using somatic mutations offers a valuable solution when other lineage-tracing methods are not applicable. Furthermore, these approaches are versatile and are capable of applying somatic mutations to binary (e.g., mutation presence/absence) and continuous (e.g., VAFs) data.
